# The Impact of an Enrichment Program on the Emirati Verbally Gifted Children

**DOI:** 10.3390/jintelligence10030068

**Published:** 2022-09-15

**Authors:** Hala Elhoweris, Najwa Alhosani, Negmeldin Alsheikh, Rhoda-Myra Garces Bacsal, Eleni Bonti

**Affiliations:** 1 Special Education Department, College of Education, United Arab Emirates University, Al Ain 112612, United Arab Emirates; 2 College of Education, United Arab Emirates University, Al Ain 112612, United Arab Emirates; 3 First Psychiatric Clinic, School of Medicine, Faculty of Health Sciences, Aristotle University of Thessaloniki, ‘Papageorgiou’ General Hospital of Thessaloniki, Agiou Pavlou 76, Pavlos Melas, 56429 Thessaloniki, Greece; 4 Department of Education, School of Education, University of Nicosia, Nicosia 2417, Cyprus

**Keywords:** verbally gifted learners, Integrated Curriculum Model (ICM), UAE, reading enrichment programs, language arts

## Abstract

Most researchers agree that verbally gifted learners should be provided with differentiated curriculum experiences that will allow them to reach their full potential. However, research is scarce in the field. The present study examined the impact of a reading enrichment program on fourth-grade students’ critical reading abilities. The program was based on the Integrated Curriculum Model (ICM). The sample consisted of forty fourth-grade verbally gifted students from a school in Dubai, who were randomly assigned to either an experimental instruction condition or a traditional instruction condition and completed pre and post-tests of language arts. A pre-and post-experimental design was used. The overall results indicated the efficacy of the differentiated enrichment program in enhancing Emirati gifted learners’ critical reading abilities. The study also provides a framework for better provision and teacher training planning regarding gifted education in the UAE.

*“Giftedness is arguably the most precious natural resource a civilization can have”*

*(Sternberg and Davidson, as cited in Pfeiffer 2002, p. 32).*

## 1. Introduction: The Impact of an Enrichment Program on the Emirati Verbally Gifted Children

### 1.1. The Concept of Giftedness

Giftedness has traditionally been conceptualized based on high performance on IQ tests. In that sense, it is often related to a commonly shared underlying, one-dimensional conception of a high intelligence quotient (IQ), often referred to as Spearman’s ‘g’ factor, which is measured by psychometric instruments (Beckmann and Minnaert 2018). The term ‘high achievement’ has also been used as a synonym for giftedness, both of which describe “students who consistently produce ideas and/or products of excellence” (Jiboye et al. 2019).

The federal Elementary and Secondary Education Act defines giftedness as: “students, children, or youth who give evidence of high achievement capability in areas such as intellectual, creative, artistic, or leadership capacity, or in specific academic fields, and who need services and activities not ordinarily provided by the school, in order to fully develop those capabilities” [ESEA, (Paul 2015)]. Several multi-dimensional models of giftedness have been developed through the years, which include various other domains or aspects of intelligence (Renzulli 1978; Monks and Mason 2000).

Furthermore, there is evidence of insufficient awareness of the definition of giftedness among teachers and parents. For instance, AlGhawi (2017) documented that, although there is an official definition of giftedness adopted by the Ministry of Education (MoE) and published for schools in Dubai, teachers and parents in the UAE continue to define giftedness partially and differently. These findings offer further testimony to the general limited knowledge around the issues of both defining and identifying giftedness, let alone applying appropriate strategic plans for gifted education.

In summary, gifted children are considered to be a heterogeneous group of students, who manifest a high ability and/or talent in several domains (e.g., cognitive/mental, linguistic, etc.), along with multiple interpersonal characteristics (Monks and Mason 2000), and who require a differentiated curriculum to meet their unique abilities and needs.

### 1.2. Verbally Gifted Students–Critical and Creative Reading

The term ‘verbally gifted’ is used to refer to children who have significantly stronger language skills than their peers (Winnebrenner 2004). These skills allow the learner to achieve a full understanding of the text being read. VanTassel-Baska (2003) defines verbally gifted as “gifted children who achieve language competency at an earlier age than their chronological age-mates” (p. 1). Verbally gifted children typically present several characteristics, which are different from their peers, related to high verbal ability, early reading, advanced vocabulary, and high-level reading comprehension (Colangelo and Davis 2003).

### 1.3. The Need for Curriculum Differentiation and Educational Modifications for the Verbally Gifted

Gifted children have different educational needs than their counterparts, which cannot be met through curricula designed for their non-gifted peers (Scruggs and Cohn 1983; Wynn 1990; Maker 2005; Wood 2008). It is critical, therefore, to identify these children and provide them with the appropriate educational programs to meet their individual needs.

Dooley (1993) cautions that a stimulating reading program for gifted readers must have at least two major components. These include provision for a quick mastering of the basic curriculum through curriculum compacting, along with a differentiated curriculum, which should involve modifications of the content and the processes used to explore that content.

Finally, Davis et al. (2011) have stressed that a lack of recognition of gifted learners’ educational and developmental needs, and a lack of appropriate accommodation provided, might put these children ‘at risk’ of failing to fully develop and flourish educationally.

## 2. Enrichment Models for Teaching Gifted Learners

The important ‘ingredients’ of instructional approaches that aid the cognitive development of gifted/talented (g/t) or high-ability students and the necessary alterations of the curriculum that should be made to enhance g/t students’ learning experiences, have been broadly recognized and extensively discussed in the literature (Brown and Campione 1994; Csikszentmihalyi et al. 1993; McLaughlin and Talbert 1993; Newstead and Wason 1995; Vye et al. 1998).

However, in many schools around the world, gifted children are given the same quantity and quality academic work as their peers. As Renzulli (2005) points out, this could be a significant waste of their school time since gifted students need to be grouped with their gifted peers for enrichment activities. Indeed, as stated by Davis et al. (2011), “grouping for enrichment, either within the class or in a resource room (pullout program), produces substantial gains in academic achievement, creativity, and other thinking skills” (p. 13).

Various enrichment models and programs have been developed and widely implemented all over the world, to facilitate and reinforce gifted students’ academic, social, creative, and thinking skills, and abilities and needs across several domains. Most of these models share an enriched view of curriculum development for the gifted, which addresses a broader conception of giftedness, taking into account principles of creativity, motivation, and independence as crucial constructs to the development of high ability. In addition, advanced process skills, such as critical thinking and creative problem solving are also viewed as central within these models (VanTassel-Baska and Brown 2007).

Some of the most well-known enrichment models include The Renzulli Schoolwide Enrichment Triad Model (SEM, Renzulli 1977; Renzulli et al. 1981) and Baska’s Integrated Curriculum Model (VanTassel-Baska 1995).

### 2.1. The Integrated Curriculum Model (ICM)

Even though most of the aforementioned enrichment models aimed at meeting gifted learners’ needs across various curriculum domains, some of these models specifically focused on promoting verbally gifted learners’ abilities, in particular (e.g., SEM, Renzulli 2005; Maker Matrix, Maker 2005).

VanTassel-Baska’s (1995) Integrated Curriculum Model (ICM) is one of the most extensively researched curriculum development models in gifted education, which also prioritizes verbal giftedness. ICM is based on differentiated instruction and includes forty units in science, language arts, social studies, and mathematics. The process of instruction and learning included in the ICM curriculum for verbally gifted students is modified in a variety of ways, including giving emphasis on higher levels of thinking and increasing the level of abstractness (Davis et al. 2011). In addition, the salient features of this curriculum include accelerated and advanced content, depth, and complexity through abstract concepts, direct study of higher-order thinking processes, interdisciplinary themes, and student research (Avery and Little 2003).

According to its developer, the ICM demonstrates the power of using a clear design approach. More precisely, its formula for a ‘successful curriculum’ is based on the fact that it couples linked subject-based standards with strong elements of differentiation for gifted learners (VanTassel-Baska and Little 2011).

VanTassel-Baska first proposed the Integrated Curriculum Model (ICM) in 1986, based on what worked with gifted learners, according to the relevant literature available at that time.

The theoretical origins of the ICM are grounded on the early conceptualizations of Vygotsky (1978), particularly on his notion of the zone of proximal development (ZPD). Other sources central to the ICM theoretical background include Csikszentmihalyi’s (1991) concept of ‘flow’, according to which gifted learners demonstrate a broader and deeper capacity to engage in learning than their typical counterparts (Csikszentmihalyi et al. 1993), and the view of interactionism, whereby the learner increases learning depth by interacting with others to enhance understanding of concepts and ideas. The theory of constructivism whereby learners construct knowledge for themselves (i.e., they are in charge of their own learning), is also central to the instructional processes applied within the ICM curriculum.

The model was further expounded upon in the subsequent years (VanTassel-Baska 2008, 2015), and today it is comprised of three interrelated dimensions that are responsive to different aspects of the gifted learner: First, it emphasizes advanced content knowledge that frames disciplines of study through the use of advanced materials in each subject area and by altering the scope and sequence of curriculum to meet the needs of the gifted (VanTassel-Baska et al. 2000). Second, it provides higher-order thinking and processing and third, it organizes learning experiences around the important aspects of a discipline, leading to an in-depth understanding of each discipline, while also providing connections across disciplines. Taken together, these relatively distinct curriculum dimensions formed the basis of the ICM, and have proven successful with gifted learners at various developmental stages and across several domain-specific areas (VanTassel-Baska and Little 2011; VanTassel-Baska and Stambaugh 2006).

### 2.2. International Research on the Application of the ICM Model

Findings from several intervention studies in different countries around the world, which have used the ICM in teaching language arts to gifted learners, provide sufficient evidence that the particular enrichment model was successful in promoting verbally gifted students’ knowledge, attitudes, motivation, and thinking skills (e.g., Brown et al. 2006; Feng et al. 2004; Gubbins et al. 2002; Kim et al. 2012; VanTassel-Baska and Brown 2007). Most of these studies used quasi-experimental research designs, which compared the pre-test/post-test performance of gifted students participating in these programs, as well as with the performance of their gifted peers, who were taught using the mainstream curriculum. Based on the results of these studies, the ICM model has demonstrated its ability and effectiveness in providing an enhanced learning experience that allows optimal learning and development of gifted learners, especially in the areas of language arts (VanTassel-Baska et al. 2009). Additional research studies suggest that the language art enrichment programs for the verbally gifted learners should include an appropriate selection of reading materials, guided critical discussions and advanced organizers for processing, the use of broad themes and concepts, independent research, and interdisciplinary connections (Winnebrenner 2004). It should be noted, however, that only a few studies have been conducted that focused only on reading instructional programs for g/t students (Wood 2008). In an earlier study, positive changes in teachers’ attitudes, student motivational response, and school district changes were documented as a result of implementing the ICM science and language arts curricula over three years (VanTassel-Baska et al. 2000). Another study conducted by Feng et al. (2004), examined the effectiveness of the ICM implementation in a suburban school district of g/t students in grades 3 to 5. Their results revealed significant levels of enhancement in gifted learners, in terms of language arts, critical reading, persuasive writing, and scientific research design skills. 

### 2.3. Verbally Gifted Student’s Education in the UAE

Gifted education has gained much popularity lately, as it has become a prominent issue in the Arabian Gulf. In response to the international calls for inclusive education as a form of equity in education, and following Merry’s (2008) suggestion that gifted students worldwide should have justice in education, the UAE educational system acknowledged that gifted children have a right to be recognized and catered to within school. Hence, the ‘School for All’ initiative, in line with the MoE’s Strategy 2010–2020, focused on encompassing all special needs services for both gifted students and students with disabilities. As a result, gifted education has been gaining momentum, interest, and support from ministries of education in these countries and government funds have been increasing. Additionally, in 2008, the MoE created the ‘development of gifted and talented students’ skills’ initiative, which was joined by many schools for gifted learners in the UAE. Hundreds of gifted students benefited from this initiative, whilst many teachers received training on identification and intervention programs for the g/t students. In 2014, the MoE introduced a new initiative called the ‘integrated system to identify and care for talents’ (AlGhawi 2017). Since then, several other organizations implemented various programs for g/t students in the Arabic Emirates (e.g., the Hamdan Bin Rashid AlMaktoum Foundation for distinguished Performance, 2015; the Emirates Association for the Gifted; etc.) and various agencies were created to support gifted education in the Gulf (e.g., the ‘Abu Dhabi Education Council’ (ADEC 2011) and the ‘Human Development Authority in Dubai’ (KHDA 2011).

In its continuous and keen efforts to excel academic performance in the country, the Ministry of Education (MoE) in the UAE launched an ambitious, strategic four-year (2017–2021), developmental plan that is wholly characterized by innovation and creativity. This aspiring education system aimed to instill a sought-after knowledge base that would produce competitiveness among society members speared at creating a distinguished realm at all venues.

The ultimate goal was to provide the UAE with the best possible human resource base, which would meet, and surpass, future market demands in various conventional and newly introduced contexts.

In addition, literacy has been and remains a cornerstone for the educational, social, economic, and personal fulfilment of UAE citizens. Indeed, according to the UAE Vision (2010), literate citizens in the UAE must be able to respond thoughtfully and articulately in oral and written forms, to fully participate in economic, political, social, and educational dialogues.

The emphasis recently given to literacy in the UAE has led educators and parents to question students’ reading and writing achievements in the English and Arabic languages. Moreover, since 2008, the United Arab Emirates have participated in several international standardized tests to examine and benchmark the performance levels of its education system. These tests include the ‘Program for International Student Assessment, (PISA) (OECD 2019, 2021), ‘Trends in International Mathematics and Science Study (TIMSS), and the ‘Progress in International Reading Literacy Study’ (PIRLS) (Martin et al. 2007).

The UAE received top ranks in the Arab world, but unfortunately, results from the PISA released in 2016, showed that UAE students continue to fall below the ‘Organization for Economic Co-operation and Development’ (OECD) average in science, reading, and mathematics (OECD 2019, 2021). This undesired result was in great conflict with the UAE’s National Agenda calls, according to which, the UAE was supposed to rank among the top 20 in PISA by 2021.

Meanwhile, there has been a virtual national ‘panic’ about reading and writing achievements in the UAE elementary, secondary, and postsecondary education. Indeed, there is a consensus that literacy levels nationally are unsatisfactory in the UAE (Ghefli 2016). This conceptualization, in combination with the discouraging results from PISA (OECD 2019), might have, however, functioned as a driving force behind reforming and improving language skills curricula with an increased focus on reading in the UAE. In addition, since reading is one of the most important skills students need to master at all academic levels, giving this topic the urgency and the absolute importance was very critical to the UAE education sector (UAE Innovation Strategy 2015).

Moreover, recent trends in critical literacy around the world have focused on critical reading and critical thinking, and the United Arab Emirates are no exception. Critical reading and critical thinking are the highest processes in reading and thinking, which entail the ability of “using careful evaluation, sound judgment, and reasoning powers” (Milan 1995, p. 218). Accordingly, the UAE emphasized the use of critical thinking and critical reading as a panacea for low language performance, as indicated by many local and international standardized tests; the impetus for that was to prepare students who can think critically, reason logically, evaluate different sources of information, and efficiently apply the learned knowledge in realistic conditions. Finally, the renewed and sustained economic growth in the United Arab Emirates and the overall well-being of all citizens in the Gulf countries led stakeholders to invest in high-quality learning (Elhoweris 2014).

Nevertheless, as AlGhawi (2017) argued, although gifted education has been adequately established in the USA and Europe (Davis and Rimm 2004), it is a relatively new initiative in the UAE. Therefore, there is a paucity of programs that address the unique educational needs of verbally gifted students. More precisely, to date, in the UAE public schools, no gifted and talented programs have been implemented that address the unique educational needs of verbally gifted students, to become productive and contributing members of society. In fact, verbally gifted students have been the most neglected group in the UAE public schools. Some of these students have not been even recognized as being gifted, because they do not write well or do not excel in all areas of language arts (AlGhawi 2017). This could be a tragic waste for them and the UAE society as well. As Davis et al. (2011) state, without appropriate education, gifted children could suffer psychological damage and permanent impairment of their abilities. Other researchers have also stressed the positive impact of challenging instruction on the emotional, affective, and social development of high-ability students (Eddles-Hirsch et al. 2010; Cross 2011).

In her study, which investigated the provision of gifted education in Dubai-UAE, using the National Association for Gifted Children (NAGC 2010) program standards and the implementation of gifted education programs in seven primary government schools in Dubai, AlGhawi (2017) revealed several shortcomings and discrepancies. More specifically, AlGhawi acknowledges that there has been a positive progression in gifted education during the 21st century; however, the findings of her recent study raised questions about the modes of implementation of gifted education in the UAE. At the same time, the results of this study highlighted additional deficiencies concerning the issues of defining and identifying giftedness.

Although previous research substantiates the need for modifications in the curriculum for gifted learners (e.g., VanTassel-Baska 2009; Winnebrenner 2004; Merry 2008), nevertheless, gifted learners in most UAE public schools are not provided with curriculum experiences that allow them to reach their full potential (AlGhawi 2017). The common practice in UAE schools to accommodate gifted children is the use of cooperative learning groupings and the use of challenging activities (Elhoweris 2014). However, this may not supply academic benefits for gifted students.

Concluding, so far, only limited research has been conducted to evaluate the effectiveness of enrichment educational models for g/t students in the UAE, which provided a direction for the current study.

## 3. Rationale for the Study

Findings from several intervention studies in different countries around the world that have used VanTassel-Baska’s enrichment model (ICM) in teaching language arts for gifted learners have proven to be successful in promoting verbally gifted students’ knowledge, attitudes, motivation, and thinking skills (Feng et al. 2004; Avery and Little 2003; VanTassel-Baska et al. 1996; VanTassel-Baska and Brown 2007, 2009).

As previously mentioned, VanTassel-Baska’s (1995) Integrated Curriculum Model (ICM), is one of the most extensively researched curriculum development models in gifted education. The model has demonstrated its ability and effectiveness in providing a learning experience that allows for optimal learning and development of gifted learners in the areas of language arts, science, and social studies (VanTassel-Baska 2009). More specifically, in the area of literacy, the curriculum effectiveness of the ICM model was assessed on US students’ literary analysis and interpretations and thinking in persuasive writing by using the four William and Mary language arts units (Feng et al. 2004; Brown et al. 2006). Several other research studies have supported the effectiveness of the use of the ICM model in the subject of language arts (VanTassel-Baska 2015).

However, although this model has proven to be effective, no study has been found that has examined the impact of such reading enrichment programs on UAE/Emirati gifted learners, especially with regards to verbally gifted learners.

Hence, the current study aimed to develop a reading enrichment program based on VanTassel-Baska’s ICM model for verbally gifted students in the UAE. The rationale for conducting this study was partially based on the scarcity of research on the area. In addition, the recent trends in critical literacy around the world, focus on critical reading and critical thinking as higher-level functioning traits (Milan 1995), along to invest in high-quality learning as a means for sustaining the renewed economic growth and the well-being of all citizens in the United Arab Emirates, also provided a solid basis for implementing the present study.

### Research Questions

The major objective of this study was to examine the impact of a reading enrichment/language arts program, based on VanTassel-Baska’s Integrated Curriculum Model, on fourth-grade verbally gifted students in the UAE.

The research questions addressed are the following:

1. To what extent are Emirati verbally gifted fourth-grade students making measurable gains in language arts when working with the differentiated enrichment model?
2. What is the impact of the differentiated enrichment model on the Emirati verbally gifted fourth-grade students’ attitudes towards learning?

## 4. Method

### 4.1. Participants–Sampling

Forty fourth-grade, nine-year-old students from two schools in the Emirate of Dubai were included in this study (20 males and 20 females). The majority of the participants were UAE nationals (90%), while 10% were from India and Iran.

An experimental group and a control group were formed according to the learning condition. Thus, participants were randomly assigned to either a language arts enrichment condition (n = 20) or a traditional instruction condition (n = 20).

The language arts enrichment program was implemented by two elementary-school Arabic language teachers, who were trained in gifted education, as well as in the basic principle of the newly developed program. Finally, four additional elementary school language teachers and three undergraduate students from the Special Education Department of the United Arab Emirates University (UAEU) were also involved in the implementation of the program and the data analysis procedure as research assistants.

### 4.2. Development of the Language Arts Enrichment Program

The newly developed enrichment program for the language arts units was created based on VanTassel-Baska’s (1995) Integrated Curriculum Model (ICM). More specifically, the language arts enrichment program for the verbally gifted learners included guided critical discussions and advanced organizers for processing, the use of broad themes and concepts, independent research, and interdisciplinary connections.

Since the ICM model emphasizes the use of advanced content, depth, and complexity through student research (VanTassel-Baska 2009), the gifted students attending the language arts enrichment condition, were provided with multiple opportunities to transfer learning from one situation to another, to perform fast processing, and inductive reasoning.

More precisely, the ICM model features three basic, interrelated dimensions. These are (1) Overarching Concepts, (2) Advance Content, and (3) Process-Product.

The Overarching Concepts are based on reading reflections that allow students to develop ideas and themes and determine integrated concepts and ideas originating from different content areas and background knowledge. The Advance Content provides gifted and average students with the opportunity to delve deeper into making synthesis across several content areas, rather than providing shallow ideas. Finally, the Process-Product allows students to explore a topic and conduct research relevant to their selected topic, or engage in a problem-based learning experience (VanTassel-Baska and Little 2011).

In developing the new language arts enrichment program, apart from the ICM’s basic dimensions, several other researchers’ suggestions were also taken into account. For example, the program involved systematic exposure to high-quality materials (as suggested by Reis et al. 2004), by grouping students based on their reading level and systematic exposure to challenging literature as a means of achieving acceleration, enrichment, as well as critical, creative and inquiry reading experiences (as proposed by Wood 2008).

An additional goal of the program was to establish critical reading and critical thinking enrichment experiences, based on a solid theoretical underpinning for assessing critical reading abilities and critical thinking in the Arabic language for the gifted fourth-grade Emirati students. More specifically, the hierarchical framework followed to design a critical reading assessment, included the levels of structural analysis, rhetoric analysis, social relevance, and holistic evaluation (Applegate et al. 2004; Huijie 2010; Poulson and Wallace 2004). This framework was expected to serve as a reading inventory, based on the theoretical construct of critical reading and critical thinking. The inventory text for guiding students’ content writing is comprised of the following components: analyzing paragraphs, discovering meaning, evaluating arguments, and responding to the text. Finally, the overall test focus inventory was accordingly designed to guide the written contents of the enrichment program.

### 4.3. Procedures and Data Analysis

The two elementary-school Arabic language teachers were trained over a week by the research team on the use of the new language arts enrichment program to implement it with the verbally gifted learners and were provided with professional development opportunities to be able to teach gifted learners.

A quasi-experimental research design was used in the study, which compared the pre-test and post-test performance of the verbally gifted students who attended the language arts enrichment program, as well as with the performance of their gifted peers, who were taught using the mainstream curriculum. This method has been commonly used in previous similar studies examining the outcomes of such programs (e.g., VanTassel-Baska 1995, 2009, 2015).

Participants completed pre-and post-tests on language arts administered by the two teachers in charge. The two teachers, along with the assistance of four other elementary school teachers and three research assistants (UAEU undergraduate students), collected the data. The measure used to assess the participants’ critical reading abilities was based on the school formal test that has been used in schools, which is related to the school curriculum. Given the specificities of the Arabic language, the language arts test is commonly used to formally assess the language abilities of primary-aged children in the Emirati countries, as it has been found to provide valid and reliable data. Hence, the reason why we chose to use the language art test for assessing the students’ critical reading abilities is that most of the other language testing assessment tools are only available in the English language, which is not the mother language of Emirati students. As several studies have shown, using formal language assessments in Arabic-speaking students might lead to significantly lower scores than proficient English speakers in reading, mathematics, and science and exams, as unfamiliar vocabulary and passive voice constructions may affect the L2 English language learners (ELLs)’ comprehension (Abedi and Lord 2001).

Furthermore, as Abedi (2002) argued, the impact of language on ELL assessment was even more obvious in content areas that had higher language demands such as reading, as ELLs may not understand complex questions, may meet unfamiliar vocabulary, and may have a slower reading pace than proficient English speakers (as cited in Ibrahim and Alhosani 2020). Other studies have also supported the use of language art school-based tests for evaluating students’ language skills, especially as a means of identifying students who are ‘at risk’, due to their language proficiency, home language, and immigrant status or due to other demographic characteristics, which is often the case among Emirati students.

Overall, school-based language arts testing has proven to be indifferent to typical, significant, demographic variables including ethnicity and family, whereas their scores also significantly predicted students’ later performance in language testing, even after controlling for multiple student and school variables (Goldschmidt and Martinez-Fernandez 2002; Wang et al. 2007).

Data were analyzed using Independent samples t-test statistical analysis to compare and evaluate pre-/post-testing results. Finally, the participating teachers were asked (through an informal short interview) to reflect upon their overall experience in implementing the language enrichment program regarding the benefits they believed it offered both to the verbally gifted students as well as to their own professional development.

To summarize, in this study we used a mixed-method approach. More specifically, quantitative data for the study was collected by using pre-/post-test scores. The qualitative data were collected by using unstructured interviews. The research assistants and the first author asked the participants open-ended questions with respect to their input about the enrichment program. In addition, teachers were also asked about their students’ attitudes toward the program. The interviews were conducted within two weeks and followed the flow of a natural conversation. According to the interviewees’ availability, the interviews were conducted in the school. These interviews were all recorded using a smartphone application. Each interview lasted for 45–60 min.

For the analysis of the interviews, the process involved a thorough review of the recordings, transcriptions, and interview notes. The coding of procedures of the interviews followed. The researchers preferred to use Microsoft Word to underline and annotate the participants’ answers in order to identify possible themes and introduce visual imagery to make it easy to recognize and relate them. During the process of coding, and analyzing the interview transcripts, several themes emerged.

The researchers used interviews to obtain in-depth data from the teachers about their attitudes toward the implementation of the enrichment program. In addition, teachers were asked about their students’ attitudes based on their own observations, which helped the researchers develop a real sense of the students’ attitudes toward the enrichment program. Finally, the interview, unlike a survey or a questionnaire, allows the interviewers to modify their questions depending on the respondents’ answers.

## 5. Results

The overall results of this study showed significant pre/post-test student gains and significant differences were revealed between the experimental and control groups in persuasive writing and literary analysis (see Tables S1 and S2 in Supplementary Materials).

To answer the first research question, the means and standard deviations of the post-test were analyzed for both the experimental and control groups. As shown in Table 1 below, the mean score in the pre-test of the control group is 32.30, which is almost equal to their mean score after administering the post-test (32.20), whereas the mean score in the pre-test of the experimental group was 32.70. After implementing the enrichment program, the mean score was 40.30, which indicates higher performance of the experimental group in the post-test.

An independent samples t-test analysis was run to determine if there were differences in the pre-test and post-test scores of the experimental group after undergoing the enrichment program. The 20 students who attended the enrichment program (M = 40.30, SD = 2.62), compared to the 20 students in the control group (M = 32.20, SD = 6.07), performed significantly better, as shown in the post-test scores, t(38) = 8.175, *p* < 0.001 (See Table 2 below).

An independent samples t-test analysis was run to determine if there were differences in the pre-test scores of the control group versus the experimental group before undergoing the enrichment program. The pre-test scores of the 20 students who attended the enrichment program (M = 40.30, SD = 2.62), compared to the pre-test scores of the 20 students in the control group (M = 32.20, SD = 6.07), were not significantly different (t(38) = 0.264, *p* = 0.793). On the other hand, the 20 students who attended the enrichment program (M = 40.30, SD = 2.62), compared to the 20 students in the control group (M = 32.20, SD = 6.07), performed significantly better, as shown by their post-test scores (t(38) = 5.483, *p* < 0.001 (See Table 3 below). Looking at the post-test mean scores of the control and experimental groups more closely, it was found that the experimental group scored significantly higher (M = 40.30, SD = 2.62) than the control group (M = 32.20, SD = 6.07). According to these results, the enrichment program had a significant positive effect on the students’ critical reading abilities, as it was evident that the Emirati fourth-grade, verbally gifted students made significant gains in language arts when working with the differentiated enrichment model. This finding provided a sufficient answer to the second research question of the study.

Concerning the participating students’ attitudes toward learning, the participating teachers reported that the experimental group students held positive attitudes towards learning and they were very engaged and interested in the differentiated enrichment model.

Finally, the participating teachers, during the informal interviews, reported that the short training experience they received during this project was considered extremely beneficial regarding their professional development, skills, and confidence in working with verbally gifted students. Moreover, the experience of implementing the language arts enrichment program helped them realize two important facts. First, they were able to witness the significant benefits of effectively providing verbally gifted students with a differentiated and challenging curriculum that met their unique needs and allowed them to reach their high-level potential. Second, they realized that teachers’ current training in gifted education is insufficient. More specifically, they claimed that the study revealed some important gaps in teachers’ and undergraduate university students’ training and professional skills, concerning various aspects of gifted education (e.g., definition/conceptualization of giftedness, assessment methods for identifying gifted learners, and familiarity with the available enrichment programs for teaching g/t students.

## 6. Discussion

To tap into the UAE’s verbally gifted children’s potential in language arts, the major objective of this study was to examine the impact of a reading enrichment program, which was developed based on VanTassel-Baska’s Integrated Curriculum Model (ICM), on fourth-grade verbally gifted students. In addition, the current study examined the participating verbally gifted students’ attitudes towards the enrichment program they attended, as well as the teachers’ views and the experiences of those involved in the project.

The overall results of the study indicated that the enrichment program had a significant positive effect on the fourth-grade Emirati verbally gifted students’ critical reading abilities, since the students made significant gains in language arts when working with the differentiated language enrichment model. Moreover, the study revealed that the gifted students held positive attitudes toward the language art enrichment model. This finding confirmed the results of previous studies, which investigated the impact of enrichment reading programs on elementary school students (e.g., Reis et al. 2008). Furthermore, the findings of these studies showed that students in the treatment groups scored statistically significantly higher than those in the control group in reading fluency, reading comprehension, and/or attitudes toward reading, which was also the case in the present study.

The overall benefits of the language arts enrichment model in promoting verbally gifted students’ knowledge, motivation, and thinking skills, as evident in our study, agree with the results of several intervention studies in different countries, which evaluated the outcomes of the ICM implementation in teaching language arts to verbally gifted learners (e.g., Brown et al. 2006; Feng et al. 2004; Kim et al. 2012; Gubbins et al. 2002; VanTassel-Baska and Brown 2007).

As mentioned earlier, the newly developed language enrichment program in this study followed the basic dimensions of the ICM, including high-quality reading materials, grouping based on reading levels, and challenging literature, along with literary analysis and persuasive writing activities. Thus, the positive post-test performance of the verbally gifted students who attended the program verified the appropriateness and effectiveness of the aforementioned ‘ingredients’ that should comprise a successful language arts enrichment program for the verbally gifted. Other researchers have also stressed the importance of including these elements in similar language art programs (e.g., Burkhalter 1995; VanTassel-Baska et al. 2002; Winnebrenner 2004; Wood 2008).

Additionally, it is important to mention that the utilization of the ICM as a baseline for developing our enrichment program was proven a successful choice, thus verifying its developer’s statement, according to which the ICM demonstrates a clear design approach since it couples linked subject-based standards with strong elements of differentiation for the gifted learners (VanTassel-Baska 2003). The positive results of our study validate that the particular model and its solid origins in Vygotsky’s theory and the ZPD concept, in particular, providing a ‘safe’ framework for designing challenging, albeit appropriate, learning experiences, which reveal gifted learners’ true potential (Burkhalter 1995). Furthermore, the results of the current study confirmed VanTassel-Baska’s (2015) claim that the ICM has proven to be a valid basis for motivating both students and teachers to think and learn at higher levels of functioning. The ease in developing our own enrichment model based on the ICM’s baselines also validated the coherence in the ICM design and its fidelity of implementation in various educational contexts, also reported by several researchers (e.g., Kim et al. 2012; Feng et al. 2004;VanTassel-Baska et al. 2002). Much earlier, VanTassel-Baska (1995) had already documented the ICM’s utility in that several school districts had successfully used the model’s baseline to develop their own curricula for gifted students.

The study also provided UAE elementary school teachers with a framework for designing and developing an appropriate curriculum for gifted learners. This finding is in line with previous findings of studies that have confirmed the positive impact of implementing such enrichment programs on teachers’ professional development and training skills as regards gifted education provision (AlGhawi 2017; Al Qarni 2010; Feng et al. 2004).

Teachers in this study stated that the whole process of developing and implementing the language arts enrichment program was a very motivating experience, which also provided them with useful skills and tools in terms of identifying, assessing, and planning the next steps of instruction for their gifted students. This finding was also in line with previous research (e.g., VanTassel-Baska 2015). More specifically, the pre- and post-test performance-based assessment procedure helped teachers to better determine their students’ general cognitive and linguistic levels and their high ability skills. Similar findings have also been documented in previous studies in the field (e.g., Kim et al. 2012; Feng et al. 2004).

Furthermore, the positive changes in both teachers’ and students’ attitudes, student motivational response, and the school district change in perspective towards giftedness that is reported in this study, were perceived as resulting from their involvement in the implementation of the language enrichment program. Respective results have been documented in similar studies following the implementation of the ICM science and language arts curricula in gifted education programs (VanTassel-Baska et al. 2000). Teachers in the current study also stated that the whole procedure helped them alter their confidence regarding their competence in differentiating the curriculum to meet the diverse needs of their g/t students. This finding is in line with Stamps’ (2004) study results, which revealed that when provided with well-designed programs and with adequate training, teachers feel confident about their skills, and therefore, are eager to alter their teaching methods and habits and differentiate the curriculum to meet the unique needs of ‘special’ students.

Withregards tothe other language teachers and the undergraduate students who also participated in the study as assistants, they reported an overall positive experience, in terms of the effectiveness of the program on their own professional development. This finding highlighted the need for regularly trained educators to acquire additional skills, knowledge, and training, to be able to support gifted education provisions. Other researchers have also stressed the general lack of professional development of mainstream teachers in terms of effectively supporting gifted learners in various countries (e.g., Hertberg-Davis and Callahan 2013), as well as in the UAE (AlGhawi 2017). The assisting teachers and undergraduate students’ roles in the project worked using co-teaching and mentoring techniques, which helped them become more skilled, knowledgeable, and aware of working with gifted students. These techniques have been previously proposed as effective forms of professional support (as alternatives to direct training) in special/gifted education contexts (Griffin et al. 2003).

### 6.1. Implications for Gifted Education Provision in the UAE

Several implications emerge from the current study’s findings that have meaning for both researchers and practitioners. The enrichment program used in this study has demonstrated its ability and effectiveness in providing a learning experience that allows for optimal learning and development of gifted learners in the areas of language arts, while it was beneficial for both students and teachers in multiple ways. Hence, the successful implementation of this model provides policymakers in the UAE with empirical data for implementing differentiated enrichment models for teaching gifted learners, which is an innovative aspect of gifted education, and which has not been previously explored in the UAE.

The above conceptualization can be considered an important message for stakeholders in the UAE, in terms of systematically considering the benefits of providing both learners and educators in gifted education with more specific and well-organized enrichment programs. A more systematic organization of such programs in the Emirates would solve several issues often recorded in the everyday gifted education practice. These include the exhibition of frustration and boredom of gifted learners due to the lack of challenging curriculums (Reis et al. 2008; Davis et al. 2011), as well as the frustration of teachers when they are asked to take over the overwhelming task of modifying the curriculum on their own to accommodate a range of diverse students’ needs, without having the necessary skills and training (Stamps 2004; AlGhawi 2017).

As previously mentioned, the education system in the UAE has recently emphasized reading as the most important skill students need to master in all academic sections (UAE Innovation Strategy 2015). In addition, the MoE has acknowledged that gifted students have a right to be recognized and catered to within the school (Merry 2008). Furthermore, the recent ‘disappointing’ results from PISA (OECD 2019), indicated that UAE students fall below the OECD average in reading, along with the worldwide emphasis given on critical and creative reading (Milan 1995). These two matters worked as incentives which positively triggered policymakers to provide verbally gifted students in the Emirati schools with more challenging and high-ability appropriate curriculums (Colangelo and Davis 2003) and to invest in high-quality learning, in general (Elhoweris 2014). However, recent research revealed various discrepancies and shortcomings regarding the practical application of the official policies and plans suggested by the MoE with regards to gifted education provision in the UAE (AlGhawi 2017; Al-Lawati 2016). More specifically, AlGhawi’s study illustrated that gifted education in the UAE is still far from effective, in terms of providing g/t learners with differentiated and challenging curriculums, as well as in terms of teacher training and awareness of the definition, identification, and provision of gifted learners, by both teachers and parents in the UAE.

Therefore, the positive results of the present study regarding the implementation of a language arts enrichment program for verbally gifted students might serve as a useful paradigm for initiating better-organized education provision for the g/t Emirati students. This can either be achieved through the already existing enrichment models (such as the ICM) or through the development of new programs, which will be designed to meet the special educational needs of each school or educational sector. Besides, as Johnsen (2006) stated, gifted education should be seen as a right, rather than a privilege. Finally, as research suggests (Bauwens et al. 1989; Hughes and Murawski 2001; Magiera and Zigmond 2005; Sileo and van Garderen 2010), apart from their proven positive effects on gifted education, such innovative enrichment programs could also be beneficial to the general field of Special Educational Needs (SEN).

Summarizing, some of the most important suggestions for improving gifted education provision, deriving from this study, as well as from previous research, can be summarized as follows: Since a distinctive gap has been detected between the national policies for gifted education developed by the UAE MoE and the actual implementation of these policies in the Emirati schools, which causes confusion among teachers, students and parents (AlGhawi 2017), there is a need for a Federal Law to formally acknowledge gifted learners as students with SEN and to safeguard their rights (Elhoweris 2014; Davis and Rimm 2004). This Law should ensure that the official policies and regulations for gifted education become mandatory for both gifted and disabled learners.

A wider and better-organized training plan for pre-service and in-service teachers in gifted education to strengthen human resources in the field, along with constant evaluation practices to improve the quality of gifted education programs (Aljughaiman et al. 2012), should become a part of the current gifted education provision in the UAE. Finally, better practices of raising awareness about giftedness among educators and parents, coupled with more systematic dissemination of policies and successful enrichment programs, should characterize gifted education provision both in the UAE and worldwide.

### 6.2. Limitations and Future Research

A limitation of the present study was its relatively small sample, which was selected from government schools only in the Emirate of Dubai. This was the mainly due to the difficulty we encountered in finding more, officially diagnosed as verbally gifted, fourth-grade children in the UAE schools. Therefore, in order to be able to generalize our findings, it is recommended that a similar study is conducted in the future that will include a much larger sample of students from various schools across the UAE and from several age groups.

Based on the findings of the current study, some suggestions for future research are the following: It is important to examine the long-term effect of this or similar enrichment programs on language arts and critical reading abilities. Furthermore, future studies should be conducted to provide more empirical data both around the issue of needs assessment and identification of the gifted learners, as well as on the development and implementation of other existing or newly designed enrichment models for meeting the needs of gifted students in various domains, including language arts, science, mathematics, etc.

In addition, the current study could be replicated within a longer period, to re-evaluate the implementation of language art enrichment programs for the verbally gifted at primary schools across the Emirates. Furthermore, since the present study was restricted to fourth-grade students, it is recommended that future similar studies could focus on the implementation of gifted programs at higher levels of school education (e.g., secondary school).

Another interesting suggestion for future research would be to implement such newly developed enrichment programs for non-gifted students in the UAE schools and assess their possible benefits to these students. Besides, there is evidence from prior research, suggesting that intervention programs with the use of the ICM or other enrichment programs were significantly effective for all students, irrespectively of giftedness (e.g., VanTassel-Baska et al. 2008; Swanson 2006).

In addition, similar studies could be replicated to evaluate the implementation of language-based enrichment programs for verbally gifted learners across different countries. Other studies could be also conducted to evaluate teachers, parents, and students’ awareness, perceptions and attitudes towards giftedness as a concept, and/or towards policies and practices of gifted education in the UAE and/or in other countries. Finally, extra research could more systematically evaluate pre-service and in-service teachers’ training and professional development in gifted education in the Emirates, to propose more effective ways of enhancing this area.

## 7. Summary and Conclusions

Gifted children constitute a heterogeneous group of students who manifest high ability and/or talent in several domains. Whether a student is gifted in reading, oral expression, or creative writing, the fact remains that gifted children differ from their peers in the way they think, both quantitatively and qualitatively. Even though giftedness, up to date, has not been captured in a single conceptualization, these differences have led several researchers to argue that gifted children have different educational needs than their counterparts, which cannot be met through curricula designed for their non-gifted peers.

Verbally gifted students, in particular, are those children who typically present higher linguistic abilities than their peers, related to high verbal ability, early reading, advanced vocabulary, and high-level reading comprehension. Therefore, researchers have suggested that, when the gifted reader enters school, instruction must go beyond the traditional basal program, and the focus of reading programs for gifted readers should be on critical and creative reading and thinking.

VanTassel-Baska’s (1995) Integrated Curriculum Model (ICM) is one of the most extensively researched curriculum development models in gifted education, which also prioritizes verbal giftedness. The process of instruction and learning included in the ICM curriculum for verbally gifted students emphasizes higher levels of thinking and increased levels of abstractness, while its salient features include accelerated and advanced content, depth, and complexity through abstract concepts, direct study of higher-order thinking processes, interdisciplinary themes, and student research.

Findings from several intervention studies in different countries around the world, that have used the ICM in teaching language arts to gifted learners, provide sufficient evidence that the particular enrichment model was successful in promoting verbally gifted students’ knowledge, attitudes, motivation, and thinking skills. Most of these studies agree on the importance of embedding higher-order skills into content and of teaching literary analysis and interpretation, along with persuasive writing, like language arts manifestations of higher-level thinking.

To date, however, no study has been found that investigated the impact of an enrichment program on the UAE verbally gifted children’s critical reading abilities. Moreover, recent research revealed various discrepancies and shortcomings regarding the practical application of the official policies and plans suggested by the MoE regarding gifted education provision in the UAE.

Hence, to fulfil UAE gifted children’s potential in reading, and to accommodate the educational needs of Emirati verbally gifted students, this study aimed to examine the impact of a reading enrichment program on fourth-grade students’ critical reading abilities. The newly developed program was based on VanTassel-Baska’s (2009) Integrated Curriculum Model ICM.

The overall results of the current study indicated that the enrichment program had a significant positive effect on the fourth-grade Emirati verbally gifted students’ critical reading abilities since the students made significant gains in language arts when working with the differentiated language enrichment model. Moreover, the study revealed that the gifted students held positive attitudes toward the language arts enrichment model.

In addition, the study provided UAE elementary school teachers with a framework for designing and developing an appropriate curriculum for gifted learners. Hence, there was a positive impact in implementing such an enrichment program on teachers’ professional development and training skills regarding gifted education provision. Furthermore, the positive changes in both teachers’ and students’ attitudes, student motivational response, professional development, and school district change of perspective towards giftedness reported in this study, were perceived as resulting from their involvement in the implementation of the language enrichment program. Additionally, the successful implementation of this model provides policymakers in the UAE with empirical data for implementing differentiated enrichment models for teaching gifted learners, which is an innovative aspect of gifted education, and which has not been previously explored in the UAE.

Concluding, a more systematic organization of such programs in the Emirates would solve several issues often recorded in the everyday gifted education practice. More precisely, a wider and better-organized training plan for pre-service and in-service teachers in gifted education to strengthen human resources in the field, along with constant evaluation practices to improve the quality of gifted education programs should become a part of the current gifted education provision in the UAE. Finally, better practices of raising awareness about giftedness among educators and parents, coupled with more systematic dissemination of policies and successful enrichment programs, should characterize gifted education provision both in the UAE and worldwide.

## Figures and Tables

**Table 1:** Descriptive Statistics.

Descriptive Statistics					
	N	Minimum	Maximum	Mean	Std. Deviation
Control-Pre-Test	20	20.00	40.00	32.30	5.95
Control-Post-Test	20	21.00	40.00	32.20	6.07
Experimental-Pre-Test	20	25.00	38.00	32.70	3.23
Experimental-PostTest	20	35.00	43.00	40.30	2.62
Valid N (listwise)	20				

**Table 2:** Independent samples t-test (within group).

Independent Samples Test (within Group)								
Groups	Test	N	Mean	Std. Deviation	t-Test	df	Level of Significance (2-Tailed)	Critical t
Control Group	Pre-Test	20	32.30	5.95	0.053	38	0.958	2.024
	Post-Test	20	32.20	6.07				
Experimental Group	Pre-Test	20	32.70	3.23	−8.175	38	0.000	
	Post-Test	20	40.30	2.62				

**Table 3:** Independent samples t-test (between groups).

Tests	Group	N	Mean	Std. Deviation	t-Test	df	Level of Significance (2-Tailed)	Critical t
Pre-Test	Control	20	32.30	5.95	0.264	38	0.793	2.024
	Experimental	20	32.70	3.23				
Post-Test	Control	20	32.20	9.07	5.483	38	0.000	
	Experimental	20	40.30	2.62				

## Data Availability

The data presented in this study are available on request from the corresponding author due to privacy issues. The data are not publicly available due to privacy.

## Supplementary Materials

The following supporting information can be downloaded at: https://www.mdpi.com/article/10.3390/jintelligence10030068/s1, Table S1: The pre-test scores (out of 45) before the implementation of the enrichment program; Table S2: The post-test scores (out of 45) after the implementation of the enrichment program.

## References

[B1-jintelligence-10-00068] Abedi Jamal (2002). Standardized Achievement Tests and English Language Learners: Psychometrics Issues. Educational Assessment.

[B2-jintelligence-10-00068] Abedi Jamal, Lord Carol (2001). The language factor in mathematics tests. Applied Measurement in Education.

[B3-jintelligence-10-00068] (2011). Abu Dhabi Education Council. https://www.thenationalnews.com/uae/abudhabi-education-council-made-a-governmentdepartment1.628636.

[B4-jintelligence-10-00068] Al Qarni Mohammed A. (2010). Evaluation of Provisions for Gifted Students in Saudi Arabia.

[B5-jintelligence-10-00068] AlGhawi Mariam A. (2017). Gifted education in the United Arab Emirates. Cogent Education.

[B6-jintelligence-10-00068] Aljughaiman Abdullah M., Ibrahim Usama M. A., Khazali Tayseer M. (2012). An evaluation of learning outcomes of summer enrichment gifted programs in Saudi Arabia. Paper presented at the Conference on Creative Education.

[B7-jintelligence-10-00068] Al-Lawati (2016). The Attitudes of GCC Citizens toward the Services Offered to Gifted Students. https://www.aaas.org/sites/default/files/BTC_Al-Lawati_E.pdf.

[B8-jintelligence-10-00068] Applegate Mary D., Quinn Kathleen B., Applegate Anthony J. (2004). Critical Reading Inventory, The: Assessing Students Reading and Thinking and Readers Passages.

[B9-jintelligence-10-00068] Avery Linda. D, Little Catherine, VanTassel-Baska Joyce, Little Catherine (2003). Concept development and learning. Content-Based Curriculum for High-Ability Learners.

[B10-jintelligence-10-00068] Bauwens Jeanne, Hourcade Jack J., Friend Marilyn (1989). Cooperative teaching: A model for general and special education integration. Remedial and Special Education.

[B11-jintelligence-10-00068] Beckmann Else, Minnaert Alexander (2018). Non-cognitive Characteristics of Gifted Students With Learning Disabilities: An In-depth Systematic Review. Frontiers in Psychology.

[B12-jintelligence-10-00068] Brown Ann L., Campione Joseph C., McGilly Kate (1994). Guided discovery in a community of learners. Classroom Lessons: Integrating Cognitive Theory and Classroom Practices.

[B13-jintelligence-10-00068] Brown Elissa, Avery Linda, Van Tassel-Baska Joyce, Worley Bess B., Stambaugh Tamra (2006). A five-state analysis of gifted education policies. Ohio policy study results. Roeper Review.

[B14-jintelligence-10-00068] Burkhalter Nancy (1995). A Vygotsky-based curriculum for teaching persuasive writing in the elementary grades. Language Arts.

[B15-jintelligence-10-00068] Colangelo Nicholas, Davis Gary A. (2003). Handbook of Gifted Education.

[B16-jintelligence-10-00068] Cross Tracy L. (2011). On the Social and Emotional Lives of Gifted Children: Understanding and Guiding Their Development.

[B17-jintelligence-10-00068] Csikszentmihalyi Mihaly (1991). Flow: The Psychology of Optimal Experience.

[B18-jintelligence-10-00068] Csikszentmihalyi Mihaly, Rathunde Kevin R., Whalen Samuel (1993). Talented Teenagers: The Roots of Success and Failure.

[B19-jintelligence-10-00068] Davis Gary, Rimm Sylvia (2004). Education of the Gifted and Talented.

[B20-jintelligence-10-00068] Davis Gary A., Rimm Sylvia B., DelSiegle (2011). Education of the Gifted and Talented.

[B21-jintelligence-10-00068] Dooley Cindy (1993). The Challenge: Meeting the Needs of Gifted Readers. Reading Teacher.

[B22-jintelligence-10-00068] Eddles-Hirsch Katrina, Vialle Wilma, Rogers Karen B., McCormick John (2010). Just challenge those high-ability learners and they’ll be all right: The impact of social context and challenging instruction on the affective development of high-ability students. Journal of Advanced Academics.

[B23-jintelligence-10-00068] Elhoweris Hala (2014). The effect of the label “Giftedness” on the United Arab Emirates pre-service teachers’ diagnosis decisions. International Journal of Education and Research.

[B24-jintelligence-10-00068] Feng Annie Xuemei, VanTassel-Baska Joyce, Quek Chwee, Bai Wenyu, O’Neill Barbara (2004). A longitudinal assessment of gifted students’ learning using the integrated curriculum model (ICM): Impacts and perceptions of the William and Mary language arts and science curriculum. Roeper Review.

[B25-jintelligence-10-00068] Ghefli Andre (2016). PIRLS 2016 Encyclopedia, The United Arab Emirates, IEA, TIMSS and PIRLS, International Study Center, Lynch School of Education, Boston College. http://pirls2016.org/.

[B26-jintelligence-10-00068] Goldschmidt Pete, Martinez-Fernandez Jose-Felipe (2002). The Relationship among Measures as Empirical Evidence of Validity: Performance Assignments, SAT-9, and High School Exit Exam Performance Incorporating Effects of School Context (CRESST Deliverable).

[B27-jintelligence-10-00068] Griffin Cynthia C., A.Winn Judith, Otis-Wilborn Amy, Kilgore Karen L. (2003). New teacher induction in special education. Executive Summary. Center on Personnel Studies in Special Education.

[B28-jintelligence-10-00068] Gubbins E. Jean, Westberg Karen L., Reis Sally M., Dinnocenti Susan T., Tieso Carol L., Muller Lisa M., Park Sunghee, Emerick Linda J., Maxfield Lori R., Burns Deborah E. (2002). Implementing a Professional Development Model Using Gifted Education Strategies with All Students.

[B29-jintelligence-10-00068] Hertberg-Davis Holly L., M.Callahan Carolyn, Hertberg-Davis Holly L., Callahan Carolyn M. (2013). Introduction. Fundamentals of Gifted Education.

[B30-jintelligence-10-00068] Hughes Claire E., Murawski Wendy A. (2001). Lessons from another field: Applying coteaching strategies to gifted education. Gifted Child Quarterly.

[B31-jintelligence-10-00068] Huijie Lin (2010). Developing a hierarchical framework of critical reading proficiency. Chinese Journal of Applied Linguistics.

[B32-jintelligence-10-00068] Ibrahim Ali, Alhosani Najwa (2020). Impact of language and curriculum on student international exam performances in the United Arab Emirates. Cogent Education.

[B33-jintelligence-10-00068] Jiboye Temitope Favour, Salaudeen Gafar Olaide, Adejumo Oluwabunmi Opeyemi, Aikomo David Olaniyi (2019). Mental ability, Self-esteem and Learning Styles as Correlate of Creativity among High Achieving Secondary School Students in Oyo State. International Journal of Innovation, Creativity and Change.

[B34-jintelligence-10-00068] Johnsen Susan K. (2006). New national standards for teachers of gifted and talented students. Tempo.

[B35-jintelligence-10-00068] Kim Kyung Hee, VanTassel-Baska Joyce, Bracken Bruce A., Feng Annie, Stambaugh Tamra, Bland Lori (2012). Project Clarion: Three years of science instruction in title I schools among K–third-grade students. Research in Science Education.

[B36-jintelligence-10-00068] (2011). Knowledge and Human Development Authority. www.khda.gov.ae.

[B37-jintelligence-10-00068] Magiera Kathleen, Zigmond Naomi (2005). Co-teaching in middle school classrooms under routine conditions: Does the instructional experience differ for students with disabilities in co-taught and solo-taught classes?. Learning Disabilities Research and Practice.

[B38-jintelligence-10-00068] Maker C. June (2005). The DISCOVER Project: Improving Assessment and Curriculum for Diverse Gifted Learners (RM05206).

[B39-jintelligence-10-00068] Martin Michael O., Mullis Ina V. S., Kennedy Ann M. (2007). Progress in International Reading Literacy Study (PIRLS): PIRLS 2006 Technical Report, International Association for the Evaluation of Educational Achievement, TIMSS and PIRLS. https://files.eric.ed.gov/fulltext/ED499436.pdf.

[B40-jintelligence-10-00068] McLaughlin Milbrey W., Talbert Joan E. (1993). Teaching for Understanding: Challenges for Policy and Practice.

[B41-jintelligence-10-00068] Merry Michael S. (2008). Educational justice and the gifted. School Field.

[B42-jintelligence-10-00068] Milan Deanne (1995). Developing Reading Skills.

[B43-jintelligence-10-00068] Monks Franz J., Mason Emanuel J., Heller Kurt A., Monks Franz J., Sternberg Robert J., Subotnik Rena F. (2000). Developmental psychology and giftedness: Theories and research. International Handbook of Giftedness and Talent.

[B44-jintelligence-10-00068] National Association for Gifted Children (2010). Redefining Giftedness for a New Century: Shifting the Paradigm. https://www.nagc.org/sites/default/files/Position%20Statement/Redefining%20Giftedness%20for%20a%20New%20Century.pdf.

[B45-jintelligence-10-00068] Newstead Stephen E., Wason Peter C. (1995). Perspectives on Thinking and Reasoning: Essays in Honor of Peter Wason.

[B46-jintelligence-10-00068] OECD (2019). PISA 2018 Assessment and Analytical Framework.

[B47-jintelligence-10-00068] OECD (2021). 21st-Century Readers: Developing Literacy Skills in a Digital World.

[B48-jintelligence-10-00068] Paul Catherine A. (2015). The Federal Elementary and Secondary Education Act.

[B49-jintelligence-10-00068] Poulson Louise, Wallace Mike, Poulson Louise, Wallace Mike (2004). Designing and writing about research: Developing a critical frame of mind. Learning to Read Critically in Teaching and Learning.

[B50-jintelligence-10-00068] Reis Sally M., Gubbins E. Jean, Briggs Christine J., Schreiber Fredric J., Richards Susannah, Jacobs Joan K., Eckert Rebecca D., Renzulli Joseph S. (2004). Reading instruction for talented readers: Case studies documenting few opportunities for continuous progress. Gifted Child Quarterly.

[B51-jintelligence-10-00068] Reis Sally M., Fogarty Elizabeth A., Eckert Rebecca D., Muller Lisa M. (2008). Schoolwide Enrichment Model Reading Framework.

[B52-jintelligence-10-00068] Renzulli Joseph S. (1977). The Enrichment Triad Model: A Guide for Developing Defensible Programs for the Gifted and Talented.

[B53-jintelligence-10-00068] Renzulli Joseph S. (1978). What makes giftedness? Re-examining a definition. Phi Delta Kappan.

[B54-jintelligence-10-00068] Renzulli Joseph S., Sternberg Robert J., Davidson Janet E. (2005). The Three-Ring Conception of Giftedness: A Developmental Model for Promoting Creative Productivity. Conceptions of Giftedness.

[B55-jintelligence-10-00068] Renzulli Joseph S., Reis Sally M., Smith Linda (1981). The Revolving-Door Model: A new way of identifying the gifted. Phi Delta Kappan.

[B56-jintelligence-10-00068] Scruggs Thomas E., Cohn Sanford J. (1983). Learning Characteristics of Verbally Gifted Students. Gifted Child Quarterly.

[B57-jintelligence-10-00068] Sileo Jane M., Garderen Delindavan (2010). Creating optimal opportunities to learn mathematics: Blending co-teaching structures with research-based practices. Teaching Exceptional Children.

[B58-jintelligence-10-00068] Stamps Lisa S. (2004). On Teaching Gifted Students: The Effectiveness of Curriculum Compacting in First Grade Classrooms. Roeper Review.

[B59-jintelligence-10-00068] Swanson Julie Dingle (2006). Breaking Through Assumptions About Low-Income, Minority Gifted Students. Gifted Child Quarterly.

[B60-jintelligence-10-00068] (2015). UAE Innovation Strategy. https://www.moei.gov.ae/assets/download/1d2d6460/National%20Innovation%20Strategy.pdf.aspx.

[B61-jintelligence-10-00068] UAE Vision (2010). United in Ambition and Determination. https://www.vision2021.ae/en/uae-vision.

[B62-jintelligence-10-00068] VanTassel-Baska Joyce (1995). The development of talent through the curriculum. Roeper Review.

[B63-jintelligence-10-00068] VanTassel-Baska Joyce (2003). Curriculum Planning and Instructional Design for Gifted Learners.

[B64-jintelligence-10-00068] VanTassel-Baska Joyce (2008). Assessment for Gifted Students.

[B65-jintelligence-10-00068] VanTassel-Baska Joyce, VanTassel-Baska Joyce L., Cross Tracy L., Olenchak F. Richard (2009). Affective curriculum and instruction for gifted learners. The Critical Issues in Equity and Excellence in Gifted Education Series. Social-Emotional Curriculum with Gifted and Talented Students.

[B66-jintelligence-10-00068] VanTassel-Baska Joyce (2015). Differentiation in action: The Integrated Curriculum Model. Revista de Educacion.

[B67-jintelligence-10-00068] VanTassel-Baska Joyce, Little Catherine (2011). Content-Based Curriculum for the Gifted.

[B68-jintelligence-10-00068] VanTassel-Baska Joyce, Brown Elissa F. (2007). Toward Best Practice: An Analysis of the Efficacy of Curriculum Models in Gifted Education. Gifted Child Quarterly.

[B69-jintelligence-10-00068] VanTassel-Baska Joyce, Brown Elissa F., Karnes Frances A., Bean Suzanne M. (2009). An analysis of gifted education curriculum models. Methods and Materials for Teaching the Gifted.

[B70-jintelligence-10-00068] VanTassel-Baska Joyce, Stambaugh Tamra (2006). Comprehensive Curriculum for the Gifted.

[B71-jintelligence-10-00068] VanTassel-Baska Joyce, Feng Annie Xuemei, Brown Elissa F., Bracken Bruce, Stambaugh Tamra, French Heather, McGowan Susan, Worley Bess, Quek Chwee, Bai Wenyu (2008). A study of differentiated instructional change over three years. Gifted Child Quarterly.

[B72-jintelligence-10-00068] VanTassel-Baska Joyce, Bracken Bruce, Feng Annie, Brown Elissa F. (2009). A longitudinal study of reading comprehension and reasoning ability of students in elementary Title I schools. Journal for the Education of the Gifted.

[B73-jintelligence-10-00068] VanTassel-Baska Joyce, Johnson Dana T., Hughes Claire E., Boyce Linda Neal (1996). A Study of Language Arts Curriculum Effectiveness with Gifted Learners. Journal for the Education of the Gifted.

[B74-jintelligence-10-00068] VanTassel-Baska Joyce, Zuo Li, Avery Linda D., Little Catherine A. (2002). A curriculum study of gifted student learning in the language arts. Gifted Child Quarterly.

[B75-jintelligence-10-00068] VanTassel-Baska Joyce, Avery Linda D., Hughes Claire E., Little Catherine A. (2000). An evaluation of the implementation of curriculum innovation: The impact of William and Mary units on schools. Journal for the Education of the Gifted.

[B76-jintelligence-10-00068] Vye Nancy J., Goldman Susan R., Voss James F., Hmelo Cindy, Williams Susan (1998). Complex mathematical problem solving by individuals and dyads. Cognition and Instruction.

[B77-jintelligence-10-00068] Vygotsky Lev S. (1978). Mind in Society: The Development of Higher Psychological Processes.

[B78-jintelligence-10-00068] Wang Jia, Niemi David, Wang Haiwen (2007). Haiwen Predictive Validity of an English Language Arts Performance Assessment, CRESST REPORT 729.

[B79-jintelligence-10-00068] Winnebrenner Susan (2004). Teaching Gifted Kids in the Regular Classroom: Strategies and Techniques Every Teacher Can Use to Meet the Academic Needs of the Gifted and Talented.

[B80-jintelligence-10-00068] Wood Patricia F. (2008). Reading Instruction With Gifted and Talented Readers: A Series of Unfortunate Events or a Sequence of Auspicious Results?. Gifted Child Today.

[B81-jintelligence-10-00068] Wynn Karen (1990). Children’s understanding of counting. Cognition.

