# Supplementary material for: The Impact of an Enrichment Program on the Emirati Verbally Gifted Children
Source: J Intell. 2022 Sep 15;10(3):68. doi: 10.3390/jintelligence10030068 (PMC9504781; doi:10.3390/jintelligence10030068)
Supplement: Supplementary file 1 [file jintelligence-10-00068-s001.zip › jintelligence-1738956-supplementary.pdf]

Table S1. The pre-test scores (*out of 45*) before the implementation of the enrichment program

**Experimental Group Students No.**	**Experimental Class**	**Control Group Students No.**	**Control Class**
Student 1	35	Student 1	31
Student 2	34	Student 2	35
Student 3	32	Student 3	30
Student 4	31	Student 4	39
Student 5	38	Student 5	24
Student 6	33	Student 6	25
Student 7	25	Student 7	30
Student 8	30	Student 8	20
Student 9	31	Student 9	23
Student 10	31	Student 10	38
Student 11	32	Student 11	30
Student 12	35	Student 12	35
Student 13	33	Student 13	32
Student 14	35	Student 14	29
Student 15	33	Student 15	33
Student 16	37	Student 16	40
Student 17	38	Student 17	39
Student 18	33	Student 18	37
Student 19	30	Student 19	38
Student 20	28	Student 20	38
**TOTAL**	**654**	**TOTAL**	**646**
MEAN	32.7	MEAN	32.3

Table S2. The post-test scores (*out of 45*) after the implementation of the enrichment program

**Experimental Group Students No.**	**Experimental Class**	**Control Group Students No.**	**Control Class**
Student 1	43	Student 1	33
Student 2	40	Student 2	38
Student 3	40	Student 3	30
Student 4	35	Student 4	40
Student 5	40	Student 5	30
Student 6	43	Student 6	23
Student 7	40	Student 7	32
Student 8	35	Student 8	21
Student 9	40	Student 9	25
Student 10	43	Student 10	40
Student 11	40	Student 11	28
Student 12	40	Student 12	40
Student 13	35	Student 13	25
Student 14	42	Student 14	30
Student 15	42	Student 15	31
Student 16	40	Student 16	30
Student 17	43	Student 17	40
Student 18	40	Student 18	35
Student 19	42	Student 19	33
Student 20	43	Student 20	40
**TOTAL**	**806**	**TOTAL**	**644**
MEAN	40.30	MEAN	32.24
